# DCD-Net: Decoupling-Centric Decomposition Network for Low-Light Image Enhancement

**DOI:** 10.3390/s25227038

**Published:** 2025-11-18

**Authors:** Wei Wang, Yi Zhu, Mingming Zhang, Chao Xie

**Affiliations:** College of Mechanical and Electronic Engineering, Nanjing Forestry University, Nanjing 210037, China; 8230310305@njfu.edu.cn (W.W.); zyds@njfu.edu.cn (Y.Z.); zhangmingming@njfu.edu.cn (M.Z.)

**Keywords:** low-light image enhancement, decomposition network, illumination and reflectance, convolutional neural networks, Transformer, Discrete Cosine Transform (DCT)

## Abstract

This paper presents a Decoupling-Centric Decomposition network for Low-Light Image Enhancement (DCD-Net). The DCDNet addresses two key challenges: (1) existing methods center on how to design the enhancement network and ignore the decomposition network’s critical role to decouple reflectance and illuminations as the first step and (2) existing decomposition networks process images directly (or with pre-denoising), ignore their compliance with the Retinex theory. Specifically, by centering on illumination–reflectance decoupling refinement, DCDNet operates without reliance on supplementary enhancement networks. It consists of a preprocessing network and a decomposition network. The preprocessing network adopts a self-supervised learning mechanism to suppress Retinex-incompatible features in the input image, thereby improving the quality of Retinex decomposition. Within the decomposition network, the reflectance net is designed to suppress the contamination of illumination on reflectance restoration by the Dual-Gated Directional Reflectance Module (DGD-RM) and Reflectance-Guided Multi-head Self-Attention (RG-MSA), while the illumination net utilizes DCT to achieve local–global illumination estimates. Comprehensive experiments were conducted on two benchmark datasets (LOL and MIT) and five unpaired datasets. The quantitative results on different datasets are as follows (measured by PSNR and SSIM): LOL v1 (20.87, 0.770) and MIT (21.66, 0.864). The average NIQE across five unpaired datasets is 3.420. Both qualitative and quantitative analyses demonstrated the superiority of DCDNet over state-of-the-art methods. Moreover, ablation studies demonstrated the effectiveness of each module in DCDNet.

## 1. Introduction

Low-light images are characterized by low brightness, low contrast, and high noise, which fail to satisfy human visual perception requirements. Critically, this degradation can severely degrade the performance of high-level computer vision tasks [1,2,3]. Therefore, low-light image enhancement (LLIE) is an important yet challenging task in computer vision. It aims to enhance visual quality by improving image contrast and restoring detail information, and to provide reliable inputs for downstream vision tasks.

**Figure 1:**
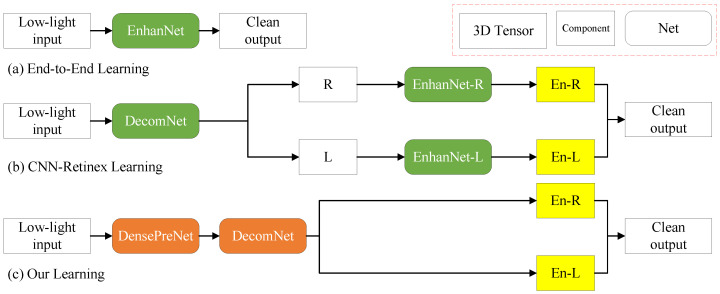
Comparison of existing learning frameworks with ours for LLIE. (**a**) End-to-end learning framework, accepted by LLNet and MBLLEN. (**b**) CNN-Retinex learning framework, accepted by Retinex-Net, KinD, URetinex-Net, and PairLIE. They require enhancement networks to optimize and refine the decomposed components after the decomposition network. (**c**) Our approach eliminates the need for additional enhancement networks by optimizing component decoupling within the decomposition network.

With the advancement of deep learning, convolutional neural networks (CNNs) have been widely applied to LLIE. Existing methods primarily fall into two categories: the first category directly learns end-to-end (Figure 1a) mappings from low-light to normal-light images using CNNs [4,5], whose data-driven nature often leads to color distortion due to the lack of physical constraints. The second category combines Convolutional Neural networks (CNNs) with Retinex theory (Figure 1b). These methods decompose an input image into illumination (*L*) and reflectance (*R*) components using dedicated decomposition networks, i.e., I=R⊙L, where ⊙ denotes element-wise multiplication. Wei et al. [6] proposed RetinexNet as a seminal work, completing the decomposition–enhancement pipeline for real-world scenes. Subsequent research has progressively optimized the enhancement network architecture to achieve better performance in LLIE tasks [7,8,9]. Some studies adopt denoising networks as an important step for images [10,11]. Zhu et al. [11] introduced RRDNet, which incorporates a noise component in addition to illumination and reflectance during decomposition. Recently, a third category of methods leveraging CNN-Transformer hybrid architectures has emerged, aiming to harness the local feature extraction prowess of CNNs and the global contextual modeling capabilities of Transformers [12,13]. However, these end-to-end methods often result in highly complex models with limited physical interpretability. Furthermore, as most employ a supervised learning paradigm, their performance is constrained by the availability of paired training data, which consequently limits their generalization capability on real-world unlabeled datasets.

Recently, Fu et al. [14] proposed the PairLIE method, introduces paired low-light images as input, innovatively leveraging the physical prior of reflectance consistency to achieve low-light image enhancement without relying on complex enhancement networks. Furthermore, it replaces the denoising network with a Retinex decomposition-adapted disturbance-removal network, providing important theoretical foundations and architectural references for our research. However, this approach exhibits two notable limitations: firstly, it fails to adequately address the critical role of the illumination component in the enhancement process; secondly, relying solely on physical priors and relatively simple network designs is insufficient for achieving comprehensive feature extraction of the reflectance component, particularly in removing illumination artifacts from it.

Despite architectural differences, these methods share a common limitation: sub-optimal constraints during decomposition lead to incomplete decoupling of illumination and reflectance. This compels heavy reliance on enhancement networks to compensate for decomposition flaws, ultimately dictating final output quality. Additionally, using denoising methods as a preprocessing step tends to cause blurring, while employing it as a postprocessing step can lead to noise amplification—both scenarios fail to adequately adhere to the adaptability required by the Retinex decomposition theory.

To address the aforementioned issues, we propose DCDNet. Our network centers on the decomposition network to optimize illumination–reflectance decoupling without relying on additional enhancement networks. It comprises two core components: (1) the Dense Preprocessing network (DPnet) and (2) a decomposition network containing a reflectance network (Rnet) and an illumination network (Lnet). DPnet leverages dense connectivity for cross-layer feature reuse. By hierarchical feature extraction, it progressively eliminates Retinex-incompatible features, thereby optimizing the subsequent decomposition efficacy. As an unsupervised model, DCDNet operates without normal-light reference images. To address this, it leverages the physical prior of reflectance consistency between paired low-light images and employs a CNN-Transformer architecture to extract reflectance features by integrating both local and global information. Rnet integrates a CNN-Transformer architecture. Its CNN stage employs a Dual-Gated Directional Reflectance Module (DGD-RM), leveraging directional convolution’s high-frequency sensitivity to mitigate the contamination of illumination on reflectance. The transformer stage utilizes Reflectance-Guided Multi-Head Self-Attention (RG-MSA) for adaptive feature restoration. LNet integrates the Discrete Cosine Transform (DCT) to introduce detailed local illumination information, thereby achieving a joint local–global illumination estimation.

In summary, the contributions of this paper are as follows:

- We propose DCDNet, an LLIE framework that eliminates additional enhancement networks by refining illumination–reflectance decoupling through its dedicated decomposition network.
- To achieve local and global feature extraction for unsupervised reflectance estimation, our Rnet leverages a CNN-Transformer hybrid network guided by the reflectance consistency prior of image pairs.
- Dense Preprocessing network (DPnet) uses a densely connected architecture for cross-layer feature reuse, optimizing Retinex decomposition. Within its Rnet component, the Dual-Gated Directional Reflectance Module (DGD-RM) mitigates illumination via directional convolution and gating. The Reflectance-Guided Multi-Head Self-Attention (RG-MSA) mechanism employs reflectance as a key cue to guide long-range feature restoration. Lnet utilizes the Discrete Cosine Transform (DCT) to introduce local illumination information to achieve a local–global illumination estimate.
- Extensive quantitative and qualitative experiments demonstrate that our final trained DCDNet surpasses existing methods across multiple datasets.

## 2. Related Works

### 2.1. Low-Light Image Enhancement

Traditional LLIE methods, including histogram equalization [15] and Retinex methods [16,17]. Histogram equalization methods enhance contrast by redistributing the brightness histogram. However, these methods are prone to causing local overexposure and artifacts. Methods based on the Retinex decompose the input image into illumination and reflectance. This is achieved by introducing various handcrafted prior constraints to optimize the decomposition process. Due to their heavy reliance on handcrafted priors and manual parameter tuning, these models suffer from poor adaptability and are prone to causing color distortion and unnatural textures [18].

In recent years, deep learning techniques have been widely applied in the field of LLIE. Several methods [6,7,8,9,19] have significantly improved enhancement quality based on a decomposition–enhancement network architecture. Wu et al. [9] proposed URetinex-Net, which models the Retinex theory as an iteratively unfoldable network structure, incorporating it as a physical prior in the deep network to guide more accurate image decomposition and enhancement. Other approaches [11,20,21,22] address the LLIE task by designing specialized decomposition networks coupled with carefully constructed loss functions. Zhu et al. [11] introduced RRDNet, which incorporates a noise component in addition to illumination and reflectance during decomposition, effectively suppressing the amplification of noise during image contrast stretching. Recently, transformers have been widely used in LLIE tasks [12,13,23,24,25,26]. Xu et al. [12] proposed an SNR-aware CNN-Transformer hybrid model, SNR-Net, for low-light image enhancement. However, its high computational complexity limits further application. Building upon this, Cai et al. [13] introduced a single-stage Retinex framework and an illumination-guided Transformer architecture, Retinexformer, which successfully applied the Transformer to the LLIE task for the first time and significantly reduced computational overhead. Nevertheless, under complex lighting conditions, existing methods often struggle to achieve a good balance between denoising and enhancement. Li et al. [26] proposed DEnet, an interpretable framework based on Discrete Cosine Transform (DCT) frequency-domain decomposition and self-supervised training with sub-image pairs. It employs a degradation-aware Transformer network to achieve integrated restoration of noise and illumination degradations in real-world low-light images. However, in real-world scenarios with severe noise or extreme illumination unevenness, the enhanced output still exhibits issues such as partial detail loss or residual noise. Unlike most previous methods that use a single low-light image as input, Fu et al. [14] were the first to propose using paired low-light images for training, a method named PairLIE, aiming to provide a general solution for low-light enhancement. However, due to its relatively simple network structure and insufficient consideration of the crucial role of illumination in the enhancement process, the method still falls short in balancing image enhancement and noise suppression, thereby limiting its overall performance.

### 2.2. Discrete Cosine Transform (DCT)

The Discrete Cosine Transform (DCT), being the core of image compression standards like JPEG, holds a foundational importance in the field of image processing. Its exceptional energy compaction property condenses image information into low-frequency and high-frequency components: low frequencies carry the fundamental structural contours and illumination information, while high frequencies contain details, textures, and noise. In recent years, researchers have deeply explored this frequency-domain prior, integrating it with deep learning to address image restoration problems [26,27,28,29,30]. Galteri et al. [27] achieved face restoration by learning to separate and process DCT components in an end-to-end manner. Li et al. [26], the DCT to separate images into different frequency components through frequency domain decomposition, constructing physical priors that guide the network to more effectively process noise and details during the enhancement process. These methods collectively corroborate that the fine-grained manipulation of high-frequency DCT and low-frequency DCT components is key to guiding models in balancing macro-structure preservation and micro-detail recovery.

## 3. Methods

In this section, we first introduce the workflow of the proposed DCDNet for LLIE, followed by detailing the loss functions of the DCDNet.

### 3.1. Network Architecture

**Figure 2:**
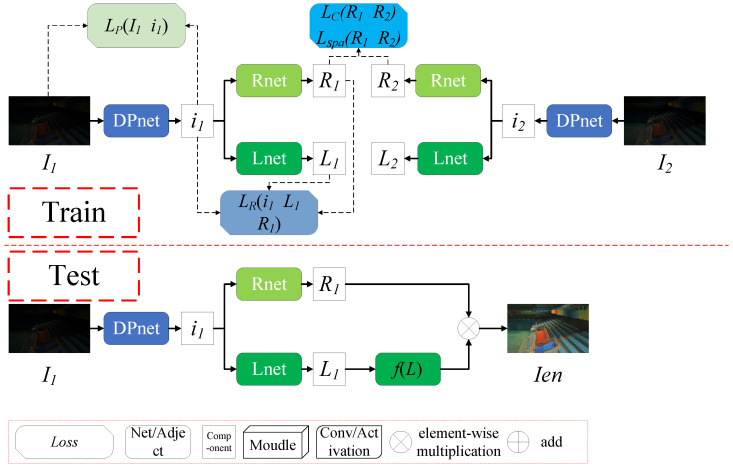
The architecture of DCDNet. During training, DPnet preprocesses low-light inputs to optimize Retinex decomposition, while Rnet and Lnet represent the reflectance and illumination decomposition networks, respectively. Optimization uses four losses: $L_{P}$ (Preprocessing), $L_{R}$ (Retinex Decomposition), $L_{Spa}$ (Spatial Consistency), and $L_{C}$ (Reflectance Consistency). During testing, trained DPnet, Lnet, and Rnet decompose inputs into reflectance and illumination; illumination is adjusted and recombined with reflectance to output enhanced images.

**Figure 3:**
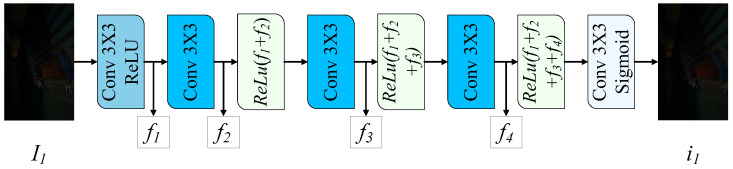
Overall Architecture of DPnet. DPnet aims to eliminate features incompatible with Retinex decomposition.

As shown in Figure 2, DCDNet consists of a Dense Preprocessing network (DPnet) and a Decomposition network. The Decomposition network consists of the Reflectance network (Rnet) and the illumination network (Lnet). DPnet aims to eliminate features incompatible with Retinex decomposition. Rnet and Lnet serve as decomposition networks to obtain the reflectance and illumination components, respectively. Table 1 illustrates the dimensional changes and primary convolutional choices of each network module. The module details will be elaborated in subsequent sections. We show the pseudocode of DCDNet in Figure 3.

In the training phase, paired low-light images $I_{1}$ and $I_{2}$ are processed by the DPnet to obtain optimized versions $i_{1}$ and $i_{2}$. These are then decomposed into illumination ($L_{1}$ and $L_{2}$) and reflectance ($R_{1}$ and $R_{2}$) using Lnet and Rnet, respectively. In the testing phase, an input low-light image *I* is processed through DCDNet to obtain the final illumination *L* and reflectance *R*. The enhanced image $I_{en}$ is then reconstructed using the following formulation:

(1) $$I_{en}=f\left(L\right)\circ R=L^{\lambda }\circ R$$

where λ is the illumination correction factor, $I_{en}$ denotes the enhanced image.

### 3.2. DCDNet Algorithm Description

The proposed DCDNet framework consists of three main components: DPnet for Retinex decomposition, Lnet for illumination estimation, and Rnet for reflectance processing. The complete algorithm proceeds as follows:

**Network Components:**

- **DPnet**: Optimizes Retinex decomposition using a dense structure
- **Lnet**: Illumination network combining DCT and CNN operations
- **Rnet**: Reflectance network incorporating DGD-RM, RG-MSA, and CNN modules

**Forward Propagation:**

1. Given input image $I_{\mathrm{input}}$, compute feature representation: $X=\mathrm{DPnet}(I_{\mathrm{input}})$
2. Estimate illumination map: L=Lnet(X)
3. Extract reflectance component: R=Rnet(X)
4. Return the triple: (X,L,R)

**DPnet Architecture:**

1. Extract initial features: $x_{1}=\mathrm{ReLU}\left({\mathrm{Conv}}_{3\times 3}\left(I\right)\right)$
2. Process with residual connections: $x_{2}=\mathrm{ReLU}\left({\mathrm{Conv}}_{3\times 3}\left(x_{1}\right)+x_{1}\right)$
3. Continue dense propagation: $x_{3}=\mathrm{ReLU}\left({\mathrm{Conv}}_{3\times 3}\left(x_{2}\right)+x_{2}+x_{1}\right)$
4. Further feature aggregation: $x_{4}=\mathrm{ReLU}\left({\mathrm{Conv}}_{3\times 3}\left(x_{3}\right)+x_{3}+x_{2}+x_{1}\right)$
5. Generate final output: $x=\mathrm{Sigmoid}({\mathrm{Conv}}_{3\times 3}\left(x_{4}\right))$

**Lnet Architecture:**

1. Apply DCT transformation: $x_{\mathrm{dct}}=\mathrm{DCT}\left(x\right)$
2. Concatenate features: $x_{\mathrm{concat}}=\mathrm{Concat}\left(x,x_{\mathrm{dct}}\right)$
3. Fuse features: $x_{\mathrm{fused}}={\mathrm{Conv}}_{1\times 1}\left(x_{\mathrm{concat}}\right)$
4. Generate illumination map: $L=\mathrm{Sigmoid}({\mathrm{CNN}}_{4-\mathrm{layer}}\left(x_{\mathrm{fused}}\right))$

**Rnet Architecture:**

1. Apply DGD-RM module: $x_{0}=\mathrm{DGD}-\mathrm{RM}\left(x\right)$
2. Process with RG-MSA: $x_{1}=\mathrm{RG}-\mathrm{MSA}\left(X,x_{0}\right)$
3. Generate reflectance map: $R=\mathrm{Sigmoid}({\mathrm{CNN}}_{3-\mathrm{layer}}\left(x_{\mathrm{relight}}\right))$

### 3.3. Dense Preprocessing Network

Serving as the preprocessing network, DPnet aims to eliminate features incompatible with Retinex decomposition, thereby optimizing the decomposition process. As shown in Figure 3, the network takes a low-light image $I\in R^{H\times W\times 3}$ as input. The network employs a densely connected architecture comprising five 3×3 convolutions for progressive feature extraction. Layers 1–4 implement cross-layer feature reuse through element-wise addition of features from all preceding layers, where each layer output $f_{i}$ is computed as follows:

(2) $$f_{i}=ReLU\left({Conv}_{i}\left(f_{i-1}\right)+\sum_{j=1}^{i-1}f_{j}\right)$$

where i=2,3,4, $f_{1}=ReLU\left({Conv}_{1}\left(I\right)\right)$. Finally, a 3×3 convolution followed by a Sigmoid function normalizes the output $i\in R^{H\times W\times 3}$ to the range [0,1].

### 3.4. Decomposition Network

The decomposition network comprises reflectance network (Rnet) and illumination network (Lnet).

#### 3.4.1. Reflectance Network

**Figure 4:**
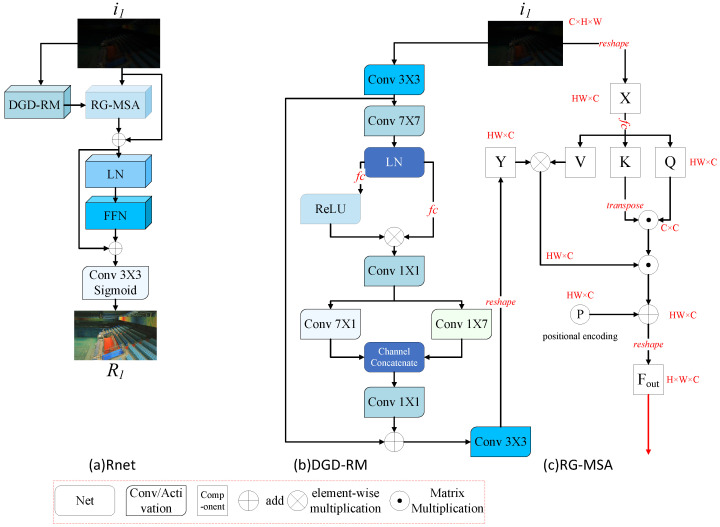
(**a**) Shows the overall architecture of Rnet, whose key components are the DGD-RM (**b**) and the RG-MSA (**c**). Detailed structures of these two components are illustrated on the right. The RG-MSA utilizes the reflectance estimated by the DGD-RM to guide the self-attention computation. Beginning with 3×3 convolution, the structure culminates in the output $F_{out}$.

As shown in Figure 4, the Rnet (Figure 4a) integrates CNN-Transformer architecture, composed of DGD-RM (Figure 4b) and RG-MSA (Figure 4c).

In DGD-RM (Figure 4b), the input features $i_{1}\in R^{H\times W\times 3}$ undergo feature extraction via 3×3 convolution, expanding the channel dimension to yield $R\in R^{H\times W\times C}$. Subsequently, a 7×7 depth-wise separable convolution extracts long-range contextual features $R_{1}\in R^{H\times W\times C}$. Two parallel 1×1 convolutions project $R_{1}$ into $R_{2}\in R^{H\times W\times 3C}$ and $R_{3}\in R^{H\times W\times 3C}$. After applying ReLU6 activation to $R_{2}$, the two features undergo element-wise multiplication. A final 1×1 convolution projects the result back to $R_{4}\in R^{H\times W\times C}$. This gating mechanism implements channel-adaptive weighting, suppressing low-frequency illumination artifacts and enhancing high-frequency reflectance through the element-wise multiplication. Subsequently, bidirectional feature compression employs dual-branch directional convolutions as follows: a 7×1 and 1×7 depth-wise separable convolution layer process the input to produce $R_{w}\in R^{H\times W\times \frac{C}{2}}$ and $R_{h}\in R^{H\times W\times \frac{C}{2}}$. These outputs are concatenated along the channel dimension and fused by a 1×1 convolution generating $R_{5}\in R^{H\times W\times C}$ to effectively suppress low-frequency illumination artifacts. To preserve ungated detail features, a residual connection adds the output features to the original input. Finally, 3×3 convolution projects the features to match the RGB channels, outputting the reflectance map $\hat{R}\in R^{H\times W\times 3}$.

RG-MSA (Figure 4c) processes spatial features as tokens for self-attention computation. The preprocessed feature map $i_{1}\in R^{H\times W\times 3}$ is reshaped into primary tokens $X\in R^{HW\times C}$. To address spatially varying reflectance distortion caused by divergent illumination, the DGD-RM mitigates illumination interference, generating refined reflectance features $\hat{R}\in R^{H\times W\times 3}$. RG-MSA leverages this reflectance information to guide region-adaptive feature restoration by aligning $\hat{R}$ with *X* through channel expansion via 1×1 convolution and reshaping into reflectance prior tokens $Y\in R^{HW\times C}$. Then *X* and *Y* are split into *k* heads as follows:

(3) $$X=[X_{1},X_{2},\ldots ,X_{k}]$$

(4) $$Y=[Y_{1},Y_{2},\ldots ,Y_{k}]$$

where $X_{i},Y_{i}\in R^{HW\times d_{k}}$, $d_{k}=\frac{c}{k}$, and i=1,2,…,k. We show the situation with k=1 and omit some details for simplification. For each $head_{i}$, three fully connected (fc) layers without bias are used to linearly project $X_{i}$ into query elements, $Q_{i}\in R^{HW\times d_{k}}$, key elements $K_{i}\in R^{HW\times d_{k}}$, and value elements $V_{i}\in R^{HW\times d_{k}}$ as follows:

(5) $$Q_{i}=X_{i}W_{Q_{i}}^{T},K_{i}=X_{i}W_{K_{i}}^{T},V_{i}=X_{i}W_{V_{i}}^{T}$$

where $W_{Q_{i}}$, $W_{K_{i}}$, and $W_{V_{i}}\in R^{d_{k}\times d_{k}}$ represent the learnable parameters of the fc layers and *T* denotes the matrix transpose. Then the self-attention for each $head_{i}$ is formulated as follows:

(6) $$\mathrm{Attention}\left(Q_{i},K_{i},V_{i},Y_{i}\right)=\left(Y_{i}⊙V_{i}\right)\mathrm{softmax}\left(\frac{K_{i}^{T}Q_{i}}{\alpha_{i}}\right)$$

where $\alpha_{i}\in R^{1}$ is a learnable scaling parameter. ⊙ is element-wise multiplication. The *k* heads are then concatenated, processed through fc layer, augmented with learnable positional encoding $P\in R^{HW\times C}$, and reshaped to output feature $F_{out}\in R^{H\times W\times C}$. Finally, the features undergo refinement through two 3×3 convolutions with ReLU activation applied only to the first layer, followed by normalization via Sigmoid function to constrain the output to the range $\left[\begin{array}{c} 0,1 \end{array}\right]$. This yields the final reflectance features $R\in R^{H\times W\times 3}$.

#### 3.4.2. Illumination Network

**Figure 5:**
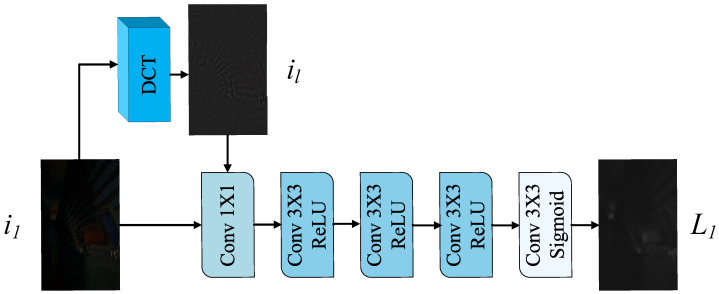
Overall architecture of Lnet. Lnet incorporates a global illumination prior through DCT.

The Lnet (Figure 5) integrates a DCT module with CNNs. Unlike existing networks that typically estimate global illumination but ignore local illumination information, Lnet extracts low-frequency features $i_{l}\in R^{H\times W\times 3}$ from the input $i_{1}\in R^{H\times W\times 3}$ using DCT, utilizing them as local illumination information compensation. These features are channel-wise concatenated with $i_{1}$, fused via 1×1 convolution, and processed through four consecutive 3×3 convolutional layers. The first three layers use ReLU activation, while the final layer employs Sigmoid activation to normalize the output $L\in R^{H\times W\times 1}$ to $\left[\begin{array}{c} 0,1 \end{array}\right]$.

### 3.5. Loss Function

In this section, we present the loss functions of DCDNet, including Retinex Loss, Reflectance Loss, and Overall Loss.

#### 3.5.1. Retinex Loss

Following Retinex theory, we decompose a low-light image into illumination and reflectance components. Inspired by PairLIE [14], we introduce two regularization losses: (1) the Preprocessing Loss $L_{P}$, which first eliminates incompatible features from the input image to optimize decomposition, and (2) the Retinex Decomposition Loss $L_{R}$ to ensure physically plausible decomposition. These losses are formulated as follows:

(7) $$L_{P}=\left|\right|I_{1}-i_{1}{\left|\right|}_{2}^{2}$$

(8) $$\begin{array}{cc} L_{R} & ={∥R\circ L-i∥}_{2}^{2}+{∥R-i/stopgrad(L)∥}_{2}^{2}\\ \; & +{∥L-L_{0}∥}_{2}^{2}+{∥\nabla L∥}_{1} \end{array}$$

where *i* denotes the preprocessed image obtained after optimization through the $L_{P}$ loss, ∇ represents the horizontal and vertical gradients. The reconstructed image is computed as $||R\circ L-{i||}_{2}^{2}$. $L_{0}$ denotes the initial illumination estimation, computed as the maximum value across RGB channels. After obtaining the illumination, the reflectance component is calculated through pixel-wise division between the preprocessed image and its illumination map, i.e., $||R-i/{stopgrad\left(L\right)||}_{2}^{2}$. stopgrad(L) represents stopping the gradient of the illumination map.

#### 3.5.2. Reflectance Loss

Spatial Consistency Loss $L_{Spa}$ encourages spatial coherence by preserving differences between adjacent regions in the low-light image pairs reflectance features.

(9) $$L_{Spa}=\frac{1}{K}\sum_{i=1}^{K}\sum_{j\in \Omega (i)}{\left(\left|\left(R_{1_{i}}-R_{1_{j}}\right)\right|-\left|\left(R_{2_{i}}-R_{2_{j}}\right)\right|\right)}^{2}$$

where *k* is the number of local regions, and $\Omega \left(i\right)$ is the four neighboring regions (top, down, left, right) centered at the region *i*. We denote $R_{1}$ and $R_{2}$ as the average intensity values of local regions within the paired reflectance maps, respectively.

Reflectance Consistency Loss $L_{C}$ is calculated based on low-light image pairs and the Retinex theory. Reflectance represents the physical properties of the image. So the low-light image pairs share the same *R*.

(10) $$L_{C}=\left|\right|R_{1}-R_{2}{\left|\right|}_{2}^{2}$$

where $R_{1}$ and $R_{2}$ refer to the reflectance components of paired low-light images. $L_{C}$ makes the network predict the same reflectance components.

#### 3.5.3. Overall Loss

The overall loss function for training our DCDNet is a liner combination of each loss as follows:

(11) $$L_{All}=w_{1}L_{P}+w_{2}L_{R}+w_{3}L_{C}+w_{4}L_{Spa}$$

where $w_{1}$, $w_{2}$, $w_{3}$, and $w_{4}$ denote the weights.

## 4. Experiments

In this section, we present the experimental setup and results of DCDNet.

### 4.1. Datasets and Metrics

We collect low-light image pairs from SICE (part2) [31] and LOL (training set) [6], which contain multi-exposure images. we collect 324 sequences (a total of 1000 low-light images). During the training phase, we randomly select two images from each sequence to form a pair. Then, we benchmark our method on the following datasets: the original LOL [6] (commonly referred to as LOL v1), its extended version LOL v2 [32] (which includes the real-captured LOL v2-real and synthetically generated LOL v2-synthetic subsets) and another 115 randomly sampled image pairs from the MITAdobe FiveK Dataset (MIT for short) as the benchmark evaluation dataset. Additionally, for no-reference evaluation, five widely accepted benchmark datasets are adopted, including MEF [33], DICM [34], LIME [35], Fusion [6], and VV [36]. For evaluation metrics, we adopt four full-reference metrics, namely the Peak Signal-to-Noise Ratio (PSNR), the Structural (SSIM) [37], the Learned Perceptual Image Patch Similarity (LPIPS) [38] and one no-reference metric, namely the Natural Image Quality Evaluator (NIQE) [39].

### 4.2. Implementation Details

We implement DCDNet with PyTorch 2.0.0 and Python 3.9.19. We use ADAM with the initial learning rate of $1\times 10^{-4}$ to optimize the network. The number of training epochs is set to 400. The learning rate is half-decayed per 100 epochs. Different datasets empirically provide different correction factors λ. The correction coefficient λ is set to 0.18 by default. For scenarios under extremely low illumination, such as those in the LOL v1 dataset, λ is set to 0.14. For the MIT dataset, which exhibits relatively adequate illumination, λ is set to 0.35. As for the hyper-parameters $w_{1}$, $w_{2}$, $w_{3}$, and $w_{4}$ in Equation (11), we set $w_{1}=500$, $w_{2}=w_{3}=w_{4}=1$. Finally, all experiments are conducted on RTX 4060 GPU.

### 4.3. Comparison Results

We compare the proposed model with state-of-the-art methods, including Retinex-Net [6], MBLLEN [5], KinD [7], URetinex-Net [9], SNR-Aware [12], Retinexformer [13], CIDNet [40], DiffLight [41], CWNet [42], RRDNet [11], EnlightenGAN [20], UEGAN [43], ZeroDCE [22], RUAS [28], SCI [44], PairLIE [14], ResQ-Net [45], and DEnet [26]. The first six methods are supervised, and the remaining methods are unsupervised together with ours.

#### 4.3.1. Qualitative Comparisons

Table 2 presents a quantitative comparison of method performance on reference-based datasets (LOL v1, LOL-v2-real, LOL-v2-synthetic, and MIT) using PSNR, SSIM, and LPIPS metrics. As an unsupervised approach, our method achieves state-of-the-art (SOTA) performance on LOL v1, LOL v2-real, and LOL v2-synthetic among unsupervised methods, and attains the best overall results on the MIT dataset.

Table 3, we evaluate method performance on five no-reference benchmarks using the NIQE metric. Our method delivers optimal or competitive results on the DICM, LIME, and MEF datasets, and achieves the best average performance across all five datasets.

Table 4 compares model complexity to assess computational efficiency. This analysis focuses specifically on deep-learning-based methods, which benefit from GPU acceleration. Our method exhibits the lowest FLOPs among all supervised approaches, while some unsupervised zero-reference methods (e.g., Zero-DCE, SCI, and ResQ-Net) naturally excel in model complexity. Our approach still surpasses several classical unsupervised methods such as EnlightenGAN, RRDNet, and DENet. Notably, compared to PairLIE—an unsupervised method with comparable performance—our method achieves comprehensive performance gains while significantly reducing model complexity, thanks to a more efficient network architecture that substantially cuts down the number of channels. This reflects our deliberate effort to strike an optimal balance between performance and efficiency. Although methods such as Zero-DCE, RUAS, and SCI exhibit lower FLOPs and Params compared to our approach, their reliance on sequential iterative optimization or recursive structures, as well as their foundation on traditional CNN architectures with heavy dependence on convolution, limits GPU parallelization, resulting in higher inference times than our method. Specifically, our DPnet enhances Retinex decomposition through densely connected convolutional modules, enabling parallel execution of Rnet and Lnet during the decomposition phase. Rnet adopts a hybrid CNN-Transformer design, where the CNN component utilizes multi-scale parallel convolutional paths to maximize parallelism. This network architecture improves computational continuity and operator fusion, thereby reducing inference time on hardware.

Additionally, as our method is built on a Transformer-based architecture, we conduct an in-depth qualitative comparison with several existing Transformer-based methods: SNR-Aware, Retinexformer, DENet, CIDNet and ours, and diffusion-based methon: DiffLight.

When compared to supervised Transformer methods such as SNR-Aware, Retinexformer, CIDNet, DENet, DiffLight and CWNet. Our approach—which follows an unsupervised paradigm and does not rely on ground-truth illumination labels during training—exhibits slightly lower performance on reference-based datasets. This outcome is consistent with the typical performance gap between supervised and unsupervised frameworks, which represents an area for future optimization. More notably, in comparison with the unsupervised method DE-net, our approach achieves superior performance across all four reference-based datasets, demonstrating clear advantages among unsupervised peers.

On non-reference datasets, our model exhibits more marked advantages; our unsupervised method achieves superior performance compared to CWNet, which exhibits the best overall performance among the supervised methods. This demonstrates the strong generalization capability of our method.

In terms of model complexity, our approach demonstrates significant efficiency advantages. On key metrics including FLOPs, number of parameters, and inference time, our method notably outperforms advanced supervised Transformer methods (such as Retinexformer, SNR-Aware, and CIDNet) as well as the unsupervised DE-net. It is particularly noteworthy that the diffusion-based DiffLight method exhibits the highest FLOPs. This efficiency advantage demonstrates that our method achieves highly competitive performance with substantially reduced computational overhead.

#### 4.3.2. Visual Comparisons

**Figure 6:**
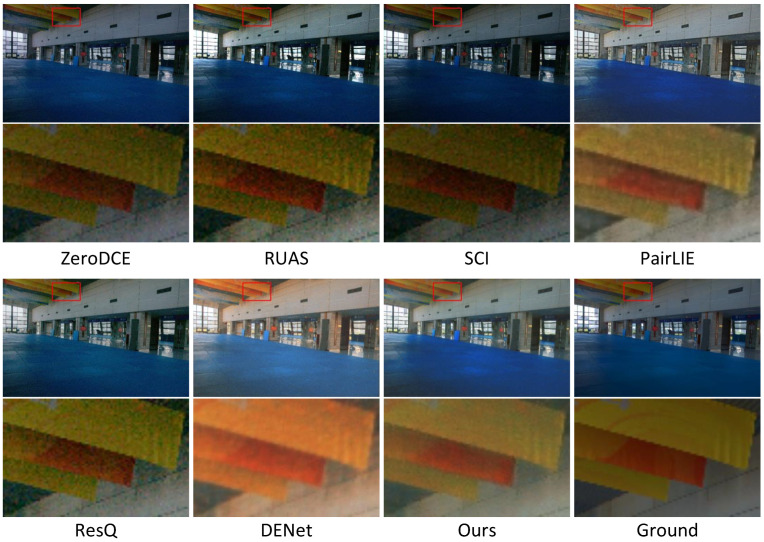
Visual comparison of the compared methods on LLIE, including ZeroDCE, RUAS, SCI, PairLIE, ResQ, DENet, and ours. The final result corresponds to the ground truth image. The dataset comes from LOL v1. The areas within the red boxes are selected for magnification, and the detailed views are presented beneath.

**Figure 7:**
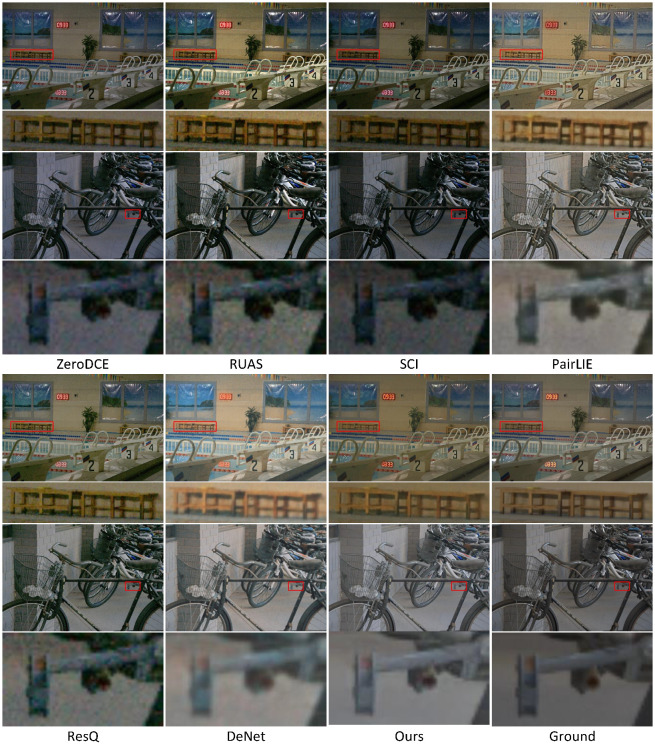
Visual comparisons of enhancement results by using different methods: ZeroDCE, RUAS, SCI, PairLIE, ResQ, DENet, and ours. The final result corresponds to the ground truth image. The dataset comes from LOL v2. The areas within the red boxes are selected for magnification, and the detailed views are presented beneath.

**Figure 8:**
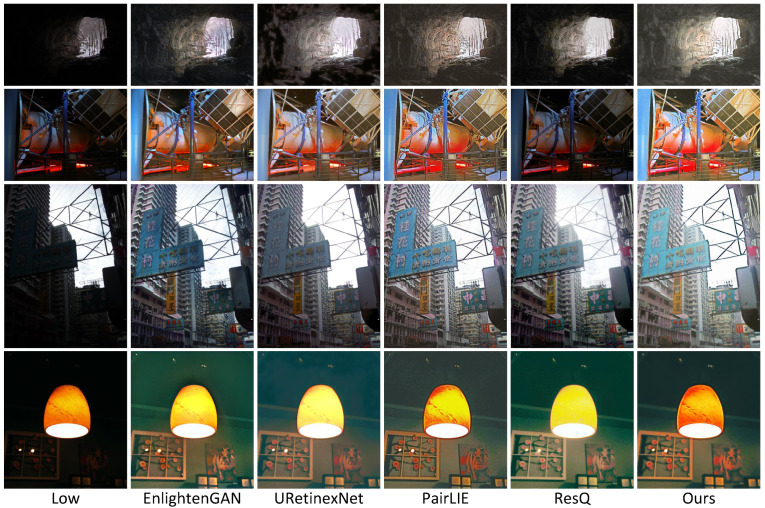
Visual comparisons of enhancement results by using different methods: EnlightenGAN, URetinexNet, PairLIE, ResQ, and ours. The datasets from top to bottom are MEF, DICM, Fusion, and LIME, respectively.

**Figure 9:**
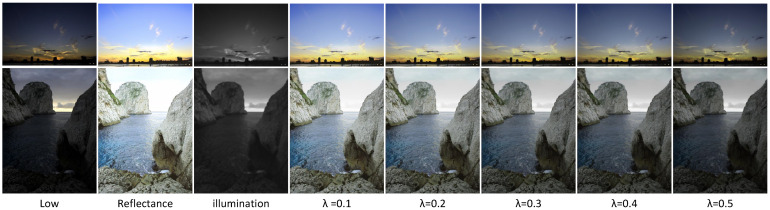
Visualization of the decomposed outputs. Enhanced results under different correction factors λ.

**Figure 10:**
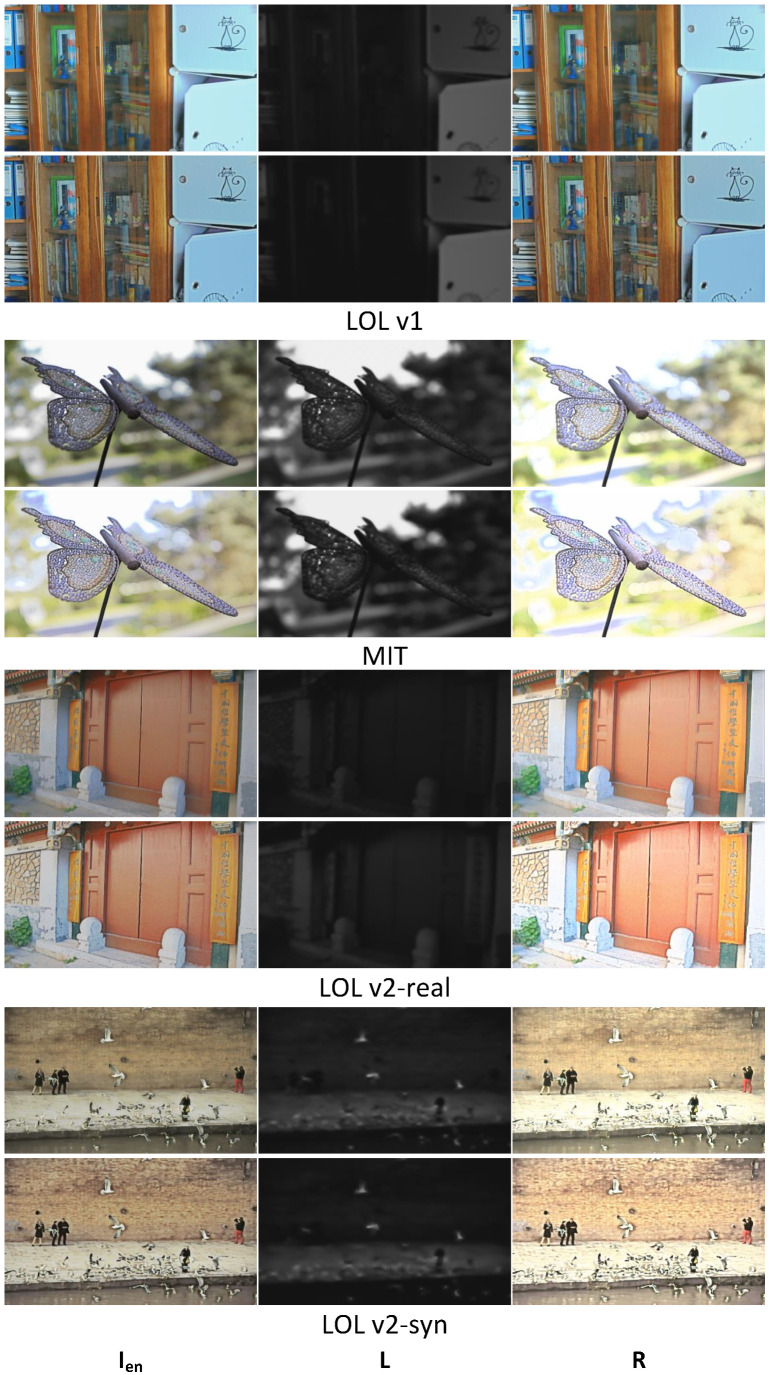
The decomposition results using our method and PairLIE. From left to right: the enhanced image ($I_{en}$), illumination (L), and reflectance (R). The dataset comes from LOL v1, MIT and LOL v2 (LOL v2-real and LOL v2-syn).

**Figure 11:**
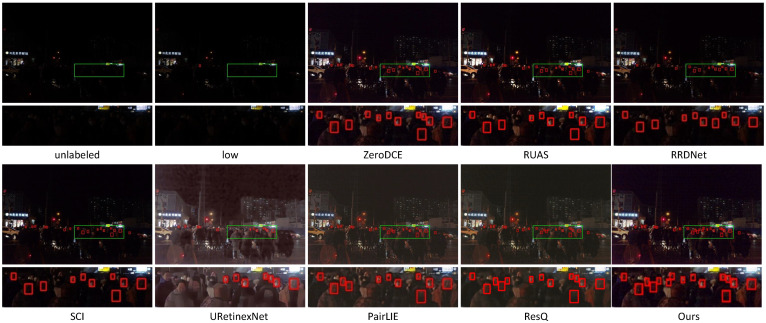
Dark face detection on a challenging example from the DARK FACE dataset, showing results from unlabeled, low, ZeroDCE, RUAS, RRDNet, SCI, URetinexNet, PairLIE, ResQ, and ours. The green boxes indicate the regions to be magnified. The enlarged views are presented below, with red boxes highlighting the detected faces.

In addition to the quantitative evaluations conducted earlier, Figure 6, Figure 7, Figure 8, Figure 9, Figure 10 and Figure 11 present visual comparisons to qualitatively compare enhancement results across different methods.

Figure 6 and Figure 7 demonstrate examples of noise suppression and detail restoration on LOL v1 and LOL v2, respectively. Although our network does not incorporate additional denoising or enhancement modules, it achieves more accurate restoration of reflectance and illumination components in low-light images through a more rational decomposition network design. Specifically, by suppressing interference from illumination information in the reflectance decomposition network, we obtain purer image reflectance. All three examples demonstrate that our method can effectively suppress noise in dark regions generated during image enhancement. Compared to other methods, our approach preserves clearer and more complete edge structures. Furthermore, in the complex scenario shown in the third row, our method not only effectively suppresses noise but also better retains critical details in the image.

Figure 8 shows examples in scenarios on unpaired datasets. We selected the top 5 methods based on the results in Table 3 for visual presentation. Most of these scenes exhibit uneven illumination distribution, thus requiring enhancement methods that combine global and local illumination information. Benefiting from our illumination network incorporating low-frequency DCT components as supplementary local illumination information, our method achieves visually superior results compared to other approaches. For example, in the third-row example, our method effectively avoids underexposure issues in the dark area of the lower-left corner. In the fourth-row example, our method successfully prevents overexposure problems in the bright areas of the light source.

Figure 9 shows the decomposed outputs of the method for different correction factors (λ). The reflectance component reveals the image’s textures and details, while the illumination component remains piecewise smooth and texture-free, indicating that our method accurately decomposes low-light images. We further present enhanced results under different correction factors. When the correction factor λ is less than 0.2, the image becomes overexposed; when λ exceeds 0.4, the image appears severely underexposed. For each dataset during the testing phase, we assign specific correction factors; for instance, λ=0.3 for the MIT dataset. The values of λ were selected to optimize enhancement performance for the specific illumination characteristics of each dataset. Users can adjust the correction factor λ during testing according to their personal preferences for image style.

Figure 10 shows the different illumination and reflectance components decomposed by our method and PairLIE. By suppressing interference from illumination information in the reflectance decomposition network, the specific performance of the reflectance maps demonstrates that, compared to PairLIE, our results contain richer textures and details while reducing potential noise-induced artifacts. In addition, our method shows advantages in characterizing both global illumination features and local detail variations. In the first two examples, our method captures local illumination details that PairLIE fails to represent, such as the books on the shelf and the stone piers at the doorway. In the next two examples, our results better align with the global characteristics of illumination, such as the light variations on the wall.

Figure 11 shows face detection in dark environments using challenging DARK FACE samples containing 28 low-illumination faces. Employing the Dual Shot Face Detector (DSFD) [46], the raw detection on this challenging sample yields very poor performance with only 1 face detected and 27 missed. URetinexNet and RRDNet detected 12 and 13 faces, respectively. ResQ, ZeroDCE, RUAS, SCI, and PairLIE detected 22, 20, 18, 18, and 19 faces, respectively. URetinexNet failed to recognize valid faces due to excessive blurriness in the enhanced images. RRDNet yielded unsatisfactory detection results because of the relatively dark enhanced images. Although ZeroDCE, RUAS, SCI, and PairLIE produced visually pleasing images, practical detection results demonstrate their ineffectiveness in detecting darker faces. Our method effectively illuminates faces in extremely dark regions while preserving details in well-lit areas, ultimately detecting 22 out of 28 faces and achieving the best detection performance, which validates the practicality of our approach.

### 4.4. Ablation Study

We conduct ablation studies to validate module effectiveness. For simplicity, evaluations use the LOL v1 dataset, and the respective performances are quantitatively evaluated using the combination of PSNR, SSIM, and LPIPS. Constrained by decomposition theory, illumination and reflectance networks remain intact. PairLIE’s [14] standard-convolution implementation serves as baseline for module-level substitution. Since RG-MSA takes the output of DGD-RM as its input, we conduct ablation studies on both modules as an integrated whole. (a) without DGD-RM and RG-MSA (in reflectance branch); (b) without DCT (in illumination branch); (c) without $L_{Spa}$ (in reflectance branch); and (d) DPnet compare to PairLIE’s Pnet (in Projection branch).

**Figure 12:**
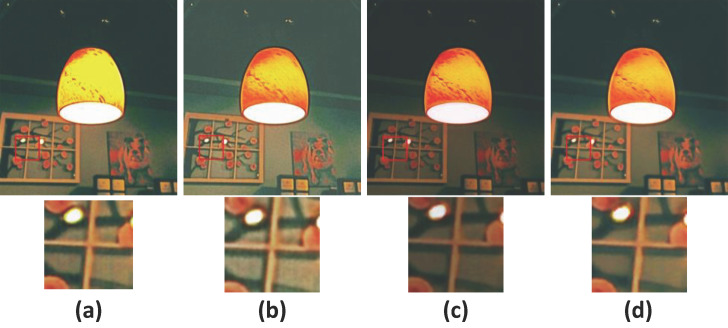
Ablation studies results on LIME dataset. (**a**) w/o DGD-RM & RG-MSA; (**b**) w/o DCT; (**c**) w/o $L_{Spa}$; (**d**) DCDNet.

Quantitative results on the LOL v1, LOL v2-real, LOL v2-syn, and MIT datasets in Table 5 confirm that each component critically enhances DCDNet’s performance. Visual results on the LIME dataset in Figure 12, removal of DGD-RM and RG-MSA induces severe color distortion in outputs due to reflectance contamination from the illumination component. When the DCT module is omitted, color reproduction remains largely intact; however, zoomed-in views reveal edge distortion around structural borders. Without $L_{Spa}$, images maintain overall naturalness but exhibit reduced contrast, particularly in background elements such as picture frames.

**Figure 13:**
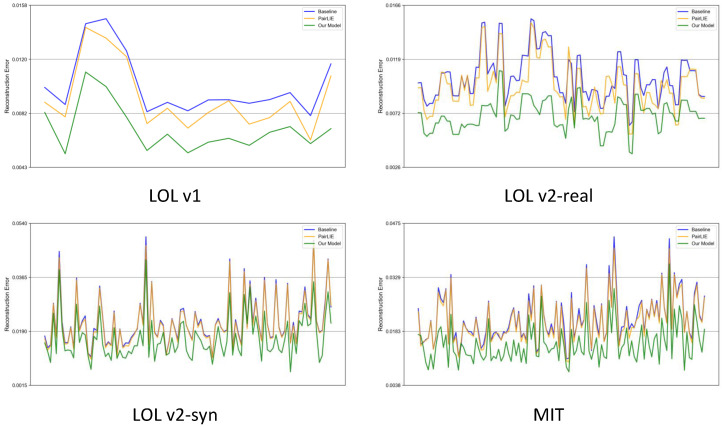
Visualization of reconstruction errors on LOL v1, LOL v2-real, LOL v2-syn, and MIT. Baseline decomposes original low-light images (blue); PairLIE uses its Pnet on projected images (orange); our method employs DPnet on projected images (green).

Figure 13 illustrate the reconstruction errors on LOL v1, LOL v2-real, LOL v2-syn, and MIT. The blue line represents decomposition using the original low-light image called baseline, i.e., |L∘R−I|; both orange and green lines denote decomposition using preprocessed images, i.e., |L∘R−i|. The orange line corresponds to the Pnet proposed in PairLIE [14], while the green line represents our DPnet. As can be seen from the figures, the reconstruction errors for the majority of images across the four datasets are lower than those of the baseline and DPnet. Table 6 quantitatively demonstrates that our average reconstruction error is also lower than that of the baseline and DPnet. Experimental results demonstrate that our DPnet not only exhibits a distinct advantage in optimizing Retinex decomposition but also outperforms the baseline and PairLIE methods in terms of both per-image optimization and the average error.

### 4.5. High-Frequency DCT and Low-Frequency DCT

There are two types of DCT: high-frequency DCT (HDCT for short) and low-frequency DCT (LDCT for short). We have experimentally validated the selection of low-frequency DCT in our DCDNet. In this part, we keep the rest of the network unchanged and separately introduce HDCT and LDCT to observe their specific contributions to the network performance.

Table 7 quantitatively compares HDCT and LDCT’s performances on reference datasets (LOL v1, LOL v2-real, LOL v2-synthetic, and MIT) using PSNR, SSIM, and LPIPS metrics. LDCT achieves the optimal performance on the LOL v1, LOL v2-real, and LOL v2-synthetic datasets, and also demonstrates superior overall performance compared to HDCT on the MIT dataset.

**Figure 14:**
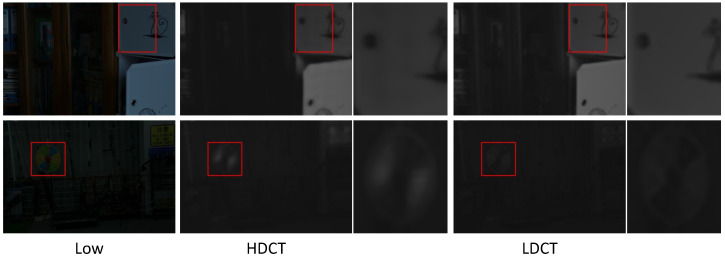
Illuminationmaps from HDCT vs. LDCT. LDCT is better. From left to right: low, HDCT, and LDCT. The red boxes indicate the regions to be magnified; the zoomed-in views are presented on the right.

**Figure 15:**
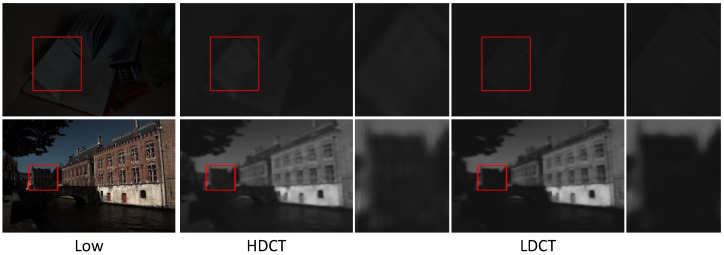
Illuminationmaps from HDCT vs. LDCT. HDCT is better. From left to right: low, HDCT, and LDCT. The red boxes indicate the regions to be magnified; the zoomed-in views are presented on the right.

Figure 14 shows that when HDCT introduces illumination reference, it additionally introduces corresponding noise, leading to illumination artifacts in the illumination component. For example, in the first row, several non-existent noise bands appear at the door, and in the second row, the partitions at the roulette wheel are ambiguous. Figure 15 demonstrates that HDCT, compared to LDCT, has advantages in local detail restoration when incorporating illumination reference. For instance, in the first row, the text on the book is clearer, and in the second row, the houses in the background are better recovered.

Experimental results indicate that both LDCT and HDCT contribute positively to image illumination estimation. LDCT is particularly effective at recovering global illumination structures, while HDCT, by leveraging high-frequency priors, shows advantages in restoring fine details. However, under the same network configuration, our comprehensive evaluation reveals that LDCT achieves overall superior performance. We hypothesize two primary reasons for this: (1) The high-frequency priors in HDCT are prone to amplifying noise, which can lead to visible artifacts in the estimated illumination. (2) The Retinex theory posits that the illumination layer primarily consists of low-frequency components, making LDCT a more physically plausible choice.

Based on this analysis, we adopt LDCT in our illumination estimation network.

### 4.6. Analysis of Failure Cases

**Figure 16:**
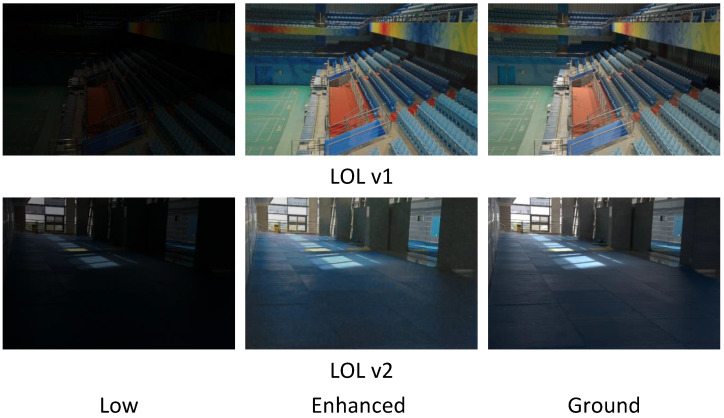
Comparison chart of failure case results. Among them, from left to right, are the low-light image, the enhanced image, and the label image in that order. The datasets come from LOL v1 and LOL v2.

To comprehensively evaluate the performance of DCDNet, we provide some failure cases and conduct an analysis. As shown in Figure 16, when processing extremely low-light areas in images, our method may exhibit localized detail loss and color deviation issues. During the training phase, our network takes paired low-light images as input and leverages the physical prior of reflectance consistency to extract reflectance through the designed Rnet. However, in extremely low-light conditions, severe noise interference leads to significant degradation of image details. Under such circumstances, using paired inputs may introduce additional degradation issues. Moreover, due to the absence of normally lit images in the training data as reference, the model struggles to effectively distinguish between noise and authentic image information. Since our DPnet is primarily designed to optimize Retinex decomposition rather than denoising, this ultimately results in the loss of local details. Regarding the color deviation issue, the Lnet incorporates local illumination information of the image through DCT, achieving local–global illumination extraction. However, in regions degraded by noise, the relative magnitudes of the RGB channel values are altered, leading to a fundamental shift in the dominant color of the image. This causes deviations in our illumination extraction method, which relies on the maximum RGB channel values, ultimately resulting in color distortion in the enhanced image.

## 5. Conclusions

This paper proposes DCDNet, an unsupervised LLIE method. Departing from the existing CNN-Retinex framework, it eliminates the need for enhancement networks through a decomposition network that refines illumination–reflectance decoupling. The preprocessing network DPNet delivers optimized inputs to decomposition network. DCT incorporates edge-structural priors during illumination estimation, while DGD-RM and RG-MSA jointly suppress illumination contamination on reflectance restoration. Extensive experiments on public benchmarks show that DCDNet outperforms the state-of-the-art methods significantly. In future work, considering that high-frequency DCT is beneficial for LLIE tasks in illumination networks, the illumination information obtained from high-frequency DCT and low-frequency DCT can be properly combined.

## Figures and Tables

**Table 1:** Network architecture specifications. The 32 indicates the use of the DGD-RM and RG-MSA modules here. The 6 indicates the use of the DCT module here.

Network	Dimensional Changes	Layer Depth	Convolution Kernel
DPnet	3 (input) — 16 — 16 — 16 — 3 (output)	4	3×3
Rnet	3 (input) — 32 — 3 — 32 — 3 (output)	4	3×3
Lnet	3 (input) — 6 — 32 — 32 — 32 — 1 (output)	5	3×3 & 1×1

**Table 2:** Conduct a quantitative evaluation of comparative methods on three reference benchmark datasets. The best and second-best results are marked in red and blue, respectively. ↑ (or ↓) means that the larger (or smaller), the better. “**Supervised**” denotes supervised methods and “**Unsupervised**” denotes unsupervised methods.

Method	LOL v1			LOL v2-Real			LOL v2-Synthetic			MIT		
	PSNR ↑	SSIM ↑	LPIPS ↓	PSNR ↑	SSIM ↑	LPIPS ↓	PSNR ↑	SSIM ↑	LPIPS ↓	PSNR ↑	SSIM ↑	LPIPS ↓
**Supervised**												
Retinex-Net [6]	14.98	0.327	0.624	16.10	0.401	0.543	14.04	0.273	0.818	14.73	0.738	0.382
MBLLEN [5]	17.86	0.627	0.225	17.87	0.694	0.272	15.78	0.501	0.599	18.01	0.751	0.283
KinD [7]	16.15	0.620	0.421	19.00	0.737	0.279	16.31	0.568	0.552	17.84	0.768	0.250
URetinex-Net [9]	17.28	0.697	0.311	21.09	0.757	0.100	17.70	0.629	0.448	18.56	0.822	0.188
SNR-Aware [12]	24.61	0.842	0.233	21.48	0.849	0.237	24.14	0.928	0.189	-	-	-
Retinexformer [13]	23.93	0.831	0.125	22.45	0.844	0.165	25.67	0.930	0.059	-	-	-
CIDNet [40]	23.81	0.870	0.086	24.11	0.867	0.116	25.12	0.939	0.045	-	-	-
DiffLight [41]	25.85	0.876	0.082	-	-	-	-	-	-	-	-	
CWNet [42]	23.60	0.849	0.065	27.39	0.900	0.038	25.50	0.936	0.020	-	-	-
**Unsupervised**												
EnlightGAN [20]	17.56	0.665	0.316	18.68	0.673	0.301	15.18	0.482	0.620	15.56	0.800	0.213
RRDNet [11]	10.16	0.406	0.483	13.70	0.508	0.290	10.50	0.310	0.729	19.60	0.830	0.229
UEGAN [43]	8.24	0.222	0.689	12.81	0.268	0.410	8.90	0.193	0.700	19.54	0.870	0.199
ZeroDCE [22]	15.33	0.567	0.335	18.46	0.580	0.305	12.99	0.365	0.684	17.96	0.843	0.226
RUAS [28]	16.15	0.462	0.368	14.89	0.455	0.372	12.19	0.297	0.748	8.46	0.540	0.585
SCI [44]	14.78	0.522	0.340	17.30	0.534	0.308	12.36	0.323	0.701	19.52	0.885	0.176
PairLIE [14]	19.51	0.736	0.248	18.23	0.735	0.260	16.11	0.566	0.560	14.47	0.759	0.198
ResQ [45]	18.28	0.596	0.323	19.67	0.573	0.347	15.65	0.394	0.687	20.87	0.915	0.107
DENet [26]	19.80	0.750	0.253	20.22	0.793	0.266	18.24	0.796	0.268	20.30	0.876	0.193
Ours	20.87	0.770	0.197	20.35	0.810	0.191	19.70	0.810	0.200	21.66	0.864	0.164

**Table 3:** Conduct quantitative evaluation of comparative methods using NIQE on five no-reference benchmark datasets. The best and second-best results are marked in red and blue, respectively.

Method	No-Reference Benchmark Dataset					Average
	**MEF**	**DICM**	**LIME**	**Fusion**	**VV**	
EnlightenGAN [20]	3.420	3.568	4.061	3.654	2.823	3.505
RRDNet [11]	3.781	6.727	6.125	5.781	2.979	5.078
UEGAN [43]	5.132	4.046	4.540	4.228	3.696	4.328
RUAS [28]	5.109	5.727	4.697	6.080	5.346	5.392
URetinexNet [9]	3.789	3.459	4.341	3.818	3.019	3.685
SNR-Aware [12]	4.180	4.712	5.742	4.323	9.872	5.765
PairLIE [14]	4.164	3.519	4.515	5.002	3.654	4.171
Retinexformer [13]	4.322	3.853	4.310	3.762	3.094	3.869
ResQ [45]	3.477	3.388	4.034	3.632	2.732	3.453
CWNet [42]	3.568	3.795	4.138	4.025	3.214	3.747
Ours	3.462	3.027	4.000	3.662	2.951	3.420

**Table 4:** Quantitative evaluation of deep-learning-based methods on model complexity in terms of Flops, Params, and inference time (Gpu seconds). “**Supervised**” denotes supervised methods and “**Unsupervised**” denotes unsupervised methods.

Method	FLOPs (G)	Params (M)	Time (S)
**Supervised**			
Retinex-Net [6]	136.02	0.8383	0.0864
MBLLEN [5]	60.02	0.4502	0.2111
KinD [7]	29.13	8.5402	0.1529
URetinex-Net [9]	58.27	0.3621	0.0176
SNR-Aware [12]	27.88	4.0132	0.0266
Retinexformer [13]	17.02	1.6057	0.0234
CIDNet [40]	7.57	1.88	-
DifLight [41]	168.3	-	-
CWNet [42]	11.3	1.23	-
**Unsupervised**			
EnlightenGAN [20]	61.01	8.6360	0.0701
RRDNet [11]	30.66	0.1282	1.5677
UEGAN [43]	32.72	16.6149	0.0435
ZeroDCE [22]	5.21	0.0789	0.0204
RUAS [28]	0.21	0.0034	0.0165
SCI [44]	0.08	0.0004	0.0160
PairLIE [14]	22.35	0.3418	0.1985
ResQ [45]	0.49	0.0123	0.0178
DENet [26]	20.40	0.312	0.0517
Ours	7.00	0.107	0.0085

**Table 5:** Ablation studies results on LOL v1, LOL v2-real, LOL v2-syn, and MIT datasets. The best results is marked in red, respectively. ↑ (or ↓) means that the larger (or smaller), the better.

Setting	LOL v1			LOL v2-Real			LOL v2-Syn			MIT		
	**PSNR** ↑	**SSIM** ↑	**LPIPS** ↓	**PSNR** ↑	**SSIM** ↑	**LPIPS** ↓	**PSNR** ↑	**SSIM** ↑	**LPIPS** ↓	**PSNR** ↑	**SSIM** ↑	**LPIPS** ↓
(a) w/o DGD-RM & RG-MSA	19.72	0.707	0.243	19.90	0.769	0.238	19.03	0.732	0.230	20.81	0.836	0.192
(b) w/o DCT	20.36	0.736	0.232	19.54	0.755	0.221	19.30	0.752	0.235	21.20	0.847	0.179
(c) w/o $L_{Spa}$	20.45	0.747	0.221	20.13	0.798	0.201	19.47	0.795	0.202	21.45	0.850	0.178
DCDNet	20.87	0.770	0.197	20.35	0.810	0.191	19.70	0.810	0.200	21.66	0.864	0.164

**Table 6:** Conduct quantitative evaluation of comparative baseline, Pnet and DPnet (ours), on four reference benchmark datasets. The better results are marked in red, respectively. ↓ means that the smaller the better.

Method	LOL v1	LOL v2-Real	LOL v2-Synthetic	MIT
	**Reconstruction Error** ↓	**Reconstruction Error** ↓	**Reconstruction Error** ↓	**Reconstruction Error** ↓
Baseline	0.0101	0.0104	0.0221	0.0217
Pnet	0.0092	0.0096	0.0216	0.0211
DPnet (Ours)	0.0070	0.0071	0.0168	0.0149

**Table 7:** Conduct quantitative evaluation of comparative HDCT and LDCT on three reference benchmark datasets. The better results are marked in red, respectively. ↑ (or ↓) means that the larger (or smaller), the better.

Method	LOL v1			LOL v2-Real			LOL v2-Synthetic			MIT		
	**PSNR** ↑	**SSIM** ↑	**LPIPS** ↓	**PSNR** ↑	**SSIM** ↑	**LPIPS** ↓	**PSNR** ↑	**SSIM** ↑	**LPIPS** ↓	**PSNR** ↑	**SSIM** ↑	**LPIPS** ↓
HDCT	20.65	0.750	0.217	20.16	0.785	0.210	19.23	0.804	0.202	21.33	0.859	0.160
LDCT (our)	20.87	0.770	0.197	20.35	0.810	0.191	19.70	0.810	0.200	21.66	0.864	0.164

## Data Availability

The raw data supporting the conclusions of this article will be made available by the authors on request.
